# Influence of Pathogen Carbon Metabolism on Interactions With Host Immunity

**DOI:** 10.3389/fcimb.2022.861405

**Published:** 2022-03-17

**Authors:** Hannah P. Berguson, Lauren W. Caulfield, Michael S. Price

**Affiliations:** ^1^ Department of Anatomical Sciences, Liberty University College of Osteopathic Medicine, Lynchburg, VA, United States; ^2^ Department of Biology and Chemistry, Liberty University, Lynchburg, VA, United States; ^3^ Department of Molecular and Cellular Sciences, Liberty University College of Osteopathic Medicine, Lynchburg, VA, United States; ^4^ Department of Medicine, Duke University School of Medicine, Durham, NC, United States

**Keywords:** mycosis, pathogenicity, host-pathogen interaction, cytokine response, macrophages (M1/M2)

## Abstract

*Cryptococcus neoformans* is a ubiquitous opportunistic fungal pathogen typically causing disease in immunocompromised individuals and is globally responsible for about 15% of AIDS-related deaths annually*. C. neoformans* first causes pulmonary infection in the host and then disseminates to the brain, causing meningoencephalitis. The yeast must obtain and metabolize carbon within the host in order to survive in the central nervous system and cause disease. Communication between pathogen and host involves recognition of multiple carbon-containing compounds on the yeast surface: polysaccharide capsule, fungal cell wall, and glycosylated proteins comprising the major immune modulators. The structure and function of polysaccharide capsule has been studied for the past 70 years, emphasizing its role in virulence. While protected by the capsule, fungal cell wall has likewise been a focus of study for several decades for its role in cell integrity and host recognition. Associated with both of these major structures are glycosylated proteins, which exhibit known immunomodulatory effects. While many studies have investigated the role of carbon metabolism on virulence and survival within the host, the precise mechanism(s) affecting host-pathogen communication remain ill-defined. This review summarizes the current knowledge on mutants in carbon metabolism and their effect on the host immune response that leads to changes in pathogen recognition and virulence. Understanding these critical interactions will provide fresh perspectives on potential treatments and the natural history of cryptococcal disease.

## 1 Introduction

*Cryptococcus neoformans* is an opportunistic fungal pathogen that primarily infects immunocompromised individuals and causes about 15% of AIDS-related deaths (Rajasingham et al., 2017). In 2006, there were an estimated 957,900 cases of cryptococcal meningitis resulting in 624,700 deaths within 3 months amongst those with HIV/AIDS (~65% mortality) (Park et al., 2009). As of 2014, global estimates of infection involving cryptococcal meningitis were estimated at 223,100 cases annually, resulting in approximately 181,100 deaths (Rajasingham et al., 2017). While incidence has decreased in the post-ART (antiretroviral therapy) era, mortality remains high for those who become infected, particularly within developing countries.

Infection by *C. neoformans* occurs through inhalation of basidiospores or desiccated yeasts which are produced from growth on plant detritus or bird droppings (Giles et al., 2009; Walsh et al., 2019). Pulmonary infection occurs first, followed by systemic dissemination including invasion of the central nervous system (CNS). Virulence factors and fitness attributes that allow *C. neoformans* to be pathogenic include thermotolerance, capsule production, melanin production, pH tolerance, and the ability to utilize multiple carbon sources. These adaptive traits assist *C. neoformans* in temporarily evading host immune responses to infection and allow persistence in immunocompromised individuals.

## 2 Virulence Factors and Fitness Attributes

### 2.1 Capsule

The capsule serves as both an offensive and defensive structure, protecting the yeast from environmental and host damage, as well as directly inhibiting host immune responses (O’meara and Alspaugh, 2012). Production of polysaccharide capsule is an essential part of *C. neoformans* virulence, making up about 25% of the total virulence composite (Mcclelland et al., 2006). With very few exceptions, acapsular strains are severely attenuated in murine infection (Fromtling et al., 1982; Chang and Kwon-Chung, 1994; O’meara et al., 2010). Production of capsule prevents desiccation and protects from oxidative stress produced by both its natural predator, the amoeba, and host phagocytic cells (Dykstra et al., 1977; Ophir and Gutnick, 1994; Steenbergen et al., 2001; Zaragoza et al., 2008; Chrisman et al., 2011). Synthesis of capsule is dynamically influenced by host-associated environmental conditions such as dehydration (Aksenov et al., 1973), neutral or alkaline pH (Zaragoza and Casadevall, 2004), high or low levels of carbon dioxide (Granger et al., 1985; Zaragoza et al., 2003) and iron deprivation (Vartivarian et al., 1993). Capsule size, composition, density, porosity and resultant immunoreactivity are variable, based on the host environment and age of the fungal cells, emphasizing the importance of morphology and fluidity to virulence within the host (Feldmesser et al., 2000; Gates et al., 2004; Charlier et al., 2005; Zaragoza et al., 2006; Cordero et al., 2011).

#### 2.1.1 Composition and Synthesis

Glucose acquisition and carbon metabolism are intrinsically related to capsule polysaccharide synthesis. Increased concentration of glucose in growth media results in increased capsule production and secretion, as does the substitution of glucose in media with mannitol, highlighting the dependence of capsule production on carbon acquisition and metabolism (Cleare and Casadevall, 1999; Guimaraes et al., 2010). In addition to the enzymes involved in synthesis and modification of these sugar donors (Griffith et al., 2004; Moyrand and Janbon, 2004), the transferases that incorporate these sugars into the polysaccharide chains of capsule are required for appropriate capsule structure and subsequent biological functions (Wills et al., 2001; Cottrell et al., 2007).

Cryptococcal capsule is composed of polysaccharide chains organized into fibers which are attached to and extending from the fungal cell wall (Cleare and Casadevall, 1999; Maxson et al., 2007b; Frases et al., 2009). Polysaccharide is secreted from the cell and is incorporated at the capsule edge (Zaragoza et al., 2006). These polysaccharide chains decrease in concentration moving outward from the cell wall, creating a somewhat permeable outer layer and a dense inner layer that prevents cell wall recognition by antibodies and complement (Gates et al., 2004; Bryan et al., 2005; Charlier et al., 2005; Maxson et al., 2007a; Maxson et al., 2007b; Frases et al., 2009; Pontes and Frases, 2015). This architectural organization confers a hydrophilic quality to the capsule which provides the ability to form a protective hydrogel or biofilm. It also contributes to the overall negative charge of the cell, which is believed to suppress phagocytosis (Nosanchuk and Casadevall, 1997; Martinez and Casadevall, 2015; Aslanyan et al., 2017; Vij et al., 2020).

The two primary polysaccharides making up this barrier are glucuronoxylomannan (GXM) and glucuronoxylomannogalactan (GXMGal), with GXM being the predominant component accounting for ~90% of polysaccharide composition (Cherniak and Sundstrom, 1994; Cherniak et al., 1998b; Mcfadden et al., 2006; Zaragoza et al., 2009). Synthesis of capsule polysaccharide involves the polymerization of simple sugars into an elongated carbohydrate chain within the cell prior to transport across the cell wall. One of the essential components of GXM is UDP-glucuronic acid, converted from UDP-glucose by UDP-glucose dehydrogenase. Mutants of *UDG1* (UDP-glucose dehydrogenase) lack any detectable capsule, as well as the ability to grow at 37°C, the normal temperature at which *C. neoformans* thrives (Griffith et al., 2004; Moyrand and Janbon, 2004). GXM is composed of an α(1,3) mannose backbone, while GXMGal is composed of an α(1,6) galactan backbone – both modified through the addition of side groups including xylose, mannose and glucuronic acid residues that form six different conformations designated as motifs 1-6 (M1-M6) (Cherniak et al., 1998b; Kozel et al., 2003; Heiss et al., 2009). These modifications alter the secondary structure of the polysaccharides, reduce molecular flexibility and create heterogeneity among different strains as well as within a population of a single strain (Todaro-Luck et al., 1989; Belay et al., 1997; Mcfadden et al., 2007).

**Figure 1:**
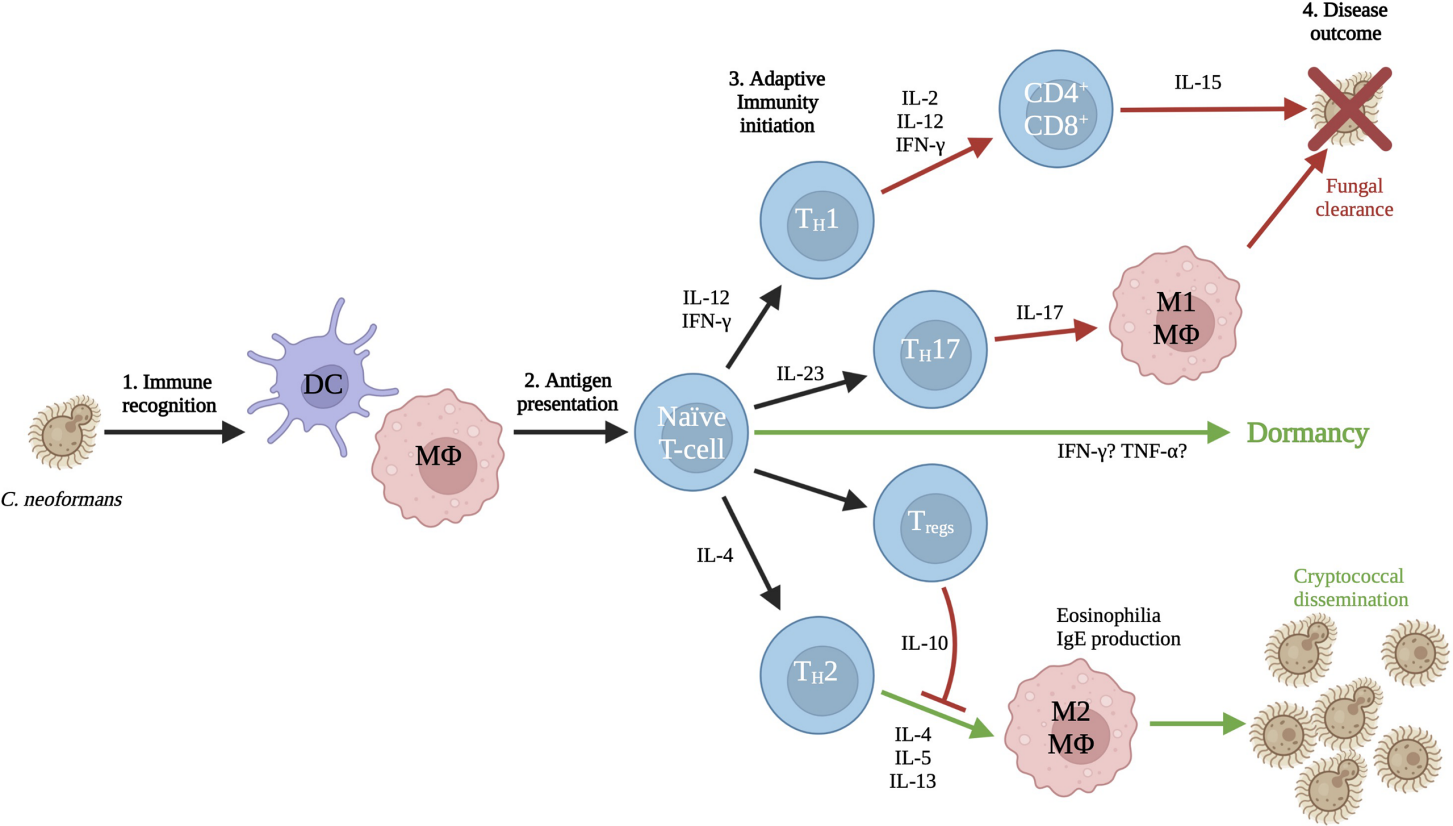
Interaction with the immune system. The first cells to interact with *C. neoformans* are dendritic cells and macrophages, particularly alveolar macrophages. These antigen presenting cells sound the alarm to naïve T-cells, who will respond by mounting either a protective or non-protective response based on the initial local cytokine environment. T-cell differentiation leads to either the incompatible M1 response by macrophages that results in clearance of the yeasts, or the compatible M2 response that results in dissemination of the yeasts to interior body sites. The cytokine responses and triggers that lead to dormancy are also as yet undetermined and an important area for future investigations. Figure adapted from (Mukaremera and Nielsen, 2017) to show possible links between host-pathogen communication and dormancy. Created with Biorender.com.

Both types of host antigen presenting cells (e.g. macrophages and dendritic cells) express pattern-recognition receptors which interact with pathogen-associated molecular-patterns (PAMPs) allowing for the generation of an immune response (Takeuchi and Akira, 2010). The toll-like receptor (TLR) family of PAMPs are membrane-bound and their binding triggers pro-inflammatory responses from within the cell including the expression of TNF-α and IFN-γ (O’mahony et al., 2008; Campuzano and Wormley, 2018). Cell-surface TLR2 and TLR4 recognize capsular polysaccharides GXM and GXMGal, while endosomal TLR9 recognizes fungal genomic DNA, specifically unmethylated CpG motifs (Nakamura et al., 2008). Activation of TLR9 results in recruitment of phagosomes and is required for elimination of the fungal pathogen by the adaptive immune system, however TLR2 and TLR4 are not involved in host defense (Nakamura et al., 2008; Ramirez-Ortiz et al., 2008; Miyazato et al., 2009; Zhang et al., 2010; Biondo et al., 2011; Qiu et al., 2012). C-type lectin receptors (CLRs) are also important for fungal recognition. These receptors also bind polysaccharides, but more specifically they can bind α-mannans (Dectin-2), mannose (CD-206/mannose receptor), mannosylated mannoproteins (DC-SIGN), and chitin (CD-206/MR) (Campuzano and Wormley, 2018). Interestingly, CD206 is a pattern recognition receptor that recognizes terminal mannose residues such as those on *C. neoformans*, and its expression is also upregulated in alternatively activated (M2) macrophages. Binding of *C. neoformans* to CD206 by these cells results in phagocytosis and subsequent intracellular growth of the yeast. The critical adaptor molecule CARD-9 responds to CLR activation and is required for M1 activation of macrophages (Campuzano et al., 2020). The resulting signaling cascade leads to M1 activation, dendritic cell maturation, and increased ROS and cytokine production (**Figure 1**).

The addition of xylose side groups among the remaining mannose residues varies between serotypes (Cherniak et al., 1980; Merrifield and Stephen, 1980). Side chains of glucuronic acid, along with the xylose residues, create two hydrophilic fringes that hide the mannan backbone from opsonization. Antigenic differences due to these substitutions and their locations have allowed for the categorization of *Cryptococcus* variations by serotype: A, B, C, D, and hybrids AD and BD (Cherniak et al., 1980; Ikeda et al., 1985), however molecular techniques have determined that serotyping alone is insufficient for determining cryptococcal species (Hagen et al., 2015). For example, the structure of *C. neoformans* serotype D has gaps between the additions of sugar groups that allow for exposure of mannan for immune recognition. In contrast, serotype A contains sugar groups arranged in a continuous band, creating an even more occluded surface against antibody binding (Kuttel et al., 2020). Urai and colleagues showed that infection with cryptococcal strains containing additional xylose, such as *C. gattii* (e.g. strain JP02) result in significantly lower immune recognition and stimulation compared to those with fewer xylose additions such as *C. neoformans* (e.g. strain H99) (Urai et al., 2015). The arrangement of acetyl groups along the mannan backbone further reduces polysaccharide flexibility and provides a structural conformation that is preferentially bound by anti-capsular antibodies (Kuttel et al., 2020). An immunostimulatory comparison of capsular polysaccharides showed O-acetylation of GXM is associated with increased recognition by antigen presenting cells as well as increased secretion of pro-inflammatory cytokines (Urai et al., 2015). Due to the lack of recognition, deacetylated mutants are more capable of evading phagocytosis resulting in hypervirulence compared to wild-type (Janbon et al., 2001). O-acetylation of capsule polysaccharides also plays an important role in virulence as it impairs chemotactic recruitment of neutrophils and their endothelial adhesion (Ellerbroek et al., 2004).

#### 2.1.2 Growth and Immune Interaction

**Figure 2:**
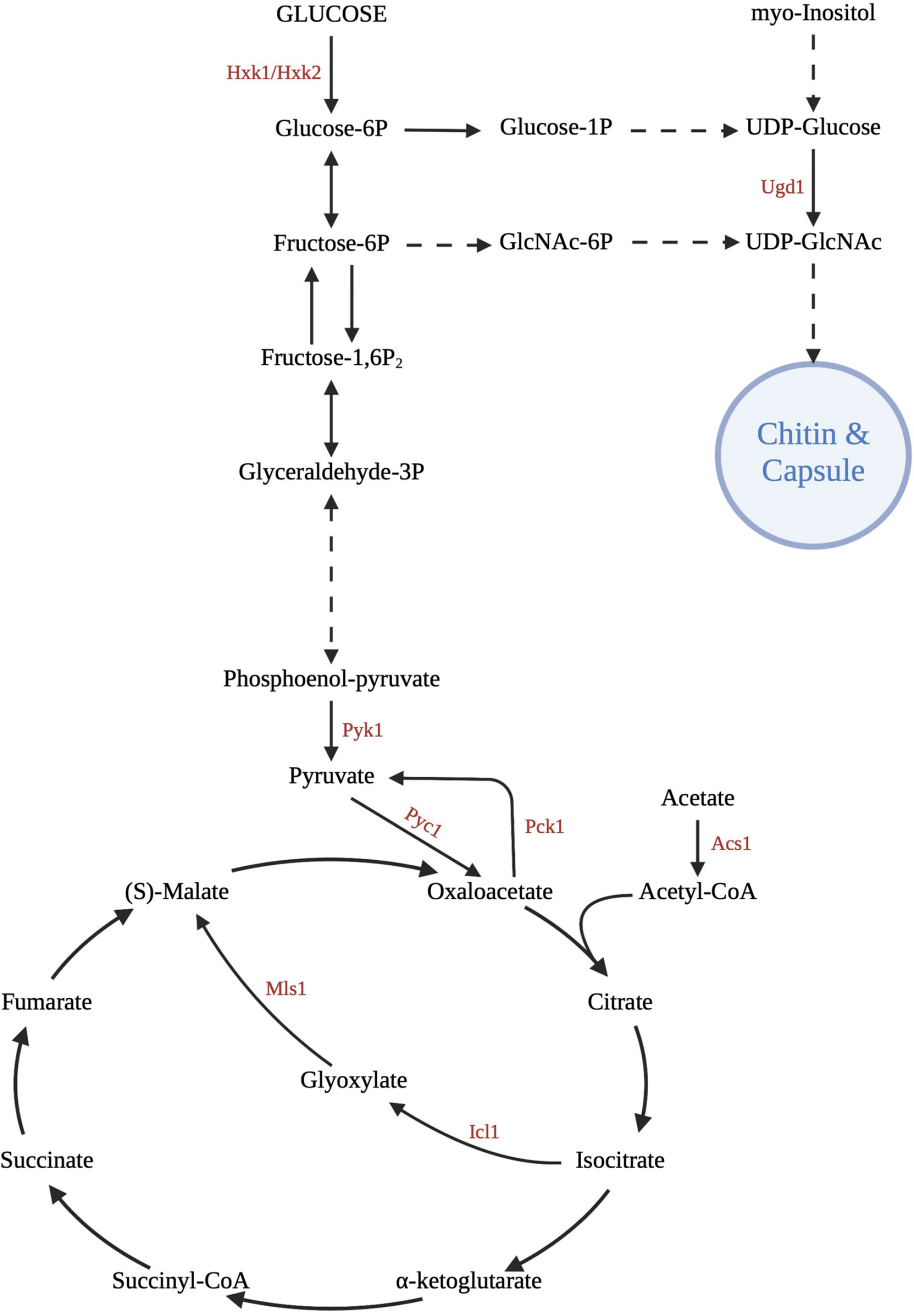
Central carbon metabolism pathways. *C. neoformans* uses glycolysis, gluconeogenesis, the tricarboxylic acid cycle, and the glyoxylate shunt for growth and survival in the host. Various gene deletion mutants have been created at important enzymatic points in these pathways to investigate the effect on virulence. Of great importance is the ability to use these intermediates and products to assemble the capsule and cell wall, major carbon-based virulence factors of *Cryptococcus*. Created with Biorender.com.

The exact mechanism of capsular growth has not been elucidated, but clearly occurs as a result of specific environmental stimuli (Zaragoza and Casadevall, 2004). Many of the capsule-inducing stressors involve nutrient deprivation, which is interesting considering the large metabolic requirements of polysaccharide accumulation (Trevijano-Contador et al., 2017). Contact with phospholipids, such as those found on macrophages or amoebae, is a trigger for capsular growth, along with other environmental stimuli that signal a threat to *Cryptococcus*, including elevated pH, CO_2_, and serum (Granger et al., 1985; Chrisman et al., 2011). Inositol catabolism has recently been shown to be essential to capsule growth and structure. Readily available inositol as a carbon source increases the size of the capsule, leading to greater virulence. Of clinical significance, inositol is abundant in the brain, allowing the yeasts to colonize this host organ (Wang et al., 2021) (**Figure 2**).

GXM has the ability to self-aggregate through divalent metal cations (such as calcium) which leads to the enlargement of capsule in a concentration dependent manner (Mcfadden et al., 2006; Nimrichter et al., 2007; Frases et al., 2008). The exopolysaccharides added during growth are different in size and mass compared to those in the steady-state capsule (Yoneda and Doering, 2008; Frases et al., 2009). In situations where capsule size must be reduced in adaptation to environmental changes, capsule does not appear to degrade, and no capsule degrading enzymes have been identified in *C. neoformans*. Instead, cells invest energy in producing buds with smaller capsules by modifying cell wall attachments of capsule during bud formation rather than degrade capsular polysaccharides (Zaragoza et al., 2009).

As the first structure to interact with the immune system, the capsule must not only be able to mitigate the immune response through structural changes, but also create a protective barrier that allows for growth and dissemination. The capsule is able to dramatically increase its size quickly after introduction of *C. neoformans* into the lungs (Feldmesser et al., 2001). Murine capsule studies have shown both size and compositional differences in capsules produced in different organs that can be attributed to the specific environments encountered in different tissues (Casadevall et al., 1992; Rivera et al., 1998; Garcia-Hermoso et al., 2004; Charlier et al., 2005). Capsular heterogeneity, even within a single population, can result in changes in immunoreactivity to specific monoclonal antibodies (Kozel et al., 2003; Garcia-Hermoso et al., 2004; Mcfadden et al., 2007). Capsule inhibits phagocytosis by monocytes in the absence of opsonization, preventing internalization of the yeast necessary for antigen presentation and subsequent T-cell activation (Vecchiarelli et al., 1994a; Vecchiarelli et al., 1994b; Syme et al., 1999; Vecchiarelli et al., 2003). Phagocytic inhibition may be due to the glucuronic acid residues in capsule imparting a negative charge across the cell surface, potentially causing an electrostatic repulsion from the host immune cells (Nosanchuk and Casadevall, 1997). Capsule structure may alter antibody binding in terms of affinity and specificity, as well as localizing antibodies deep within the capsule, reducing opsonization effectiveness. Additionally, *C. neoformans* also has been shown to metabolize opsonins through degradation by secreted proteases (Chen et al., 1996). Once opsonized and phagocytosed, capsule protects the yeast from oxidative damage (Zaragoza et al., 2008) while also interfering with the maintenance of pH within the phagolysosome (De Leon-Rodriguez et al., 2018). The phagolysosomal membrane may be maintained, releasing *C. neoformans via* non-lytic phagosomal extrusion, or it may rupture, releasing yeasts into the cytosol where they can replicate undetected (Alvarez and Casadevall, 2006; Ma et al., 2006; Voelz and May, 2010).

Unlike macrophages, dendritic cells do not require activation for *C. neoformans* killing and can quickly kill yeasts within the phagolysosome through oxidative and non-oxidative mechanisms (Wozniak et al., 2006). Additionally, they are significantly more efficient in antigen presentation to T-cells (Syme et al., 2002). Cryptococcal antigens (e.g. mannoproteins) induce IL-12 and TNF-α secretion in dendritic cells, promoting a protective T_H_1 response (Pietrella et al., 2005). However, the extracellular environment during cryptococcal infection naturally induces a non-protective T_H_2 response, including increasing the accumulation of immature dendritic cells in proximal lymph nodes (Osterholzer et al., 2009).

Encapsulated yeasts as well as purified capsular polysaccharides have been shown to inhibit the expression of host cytokines and chemokines (Lupo et al., 2008). Extracellular *C. neoformans* alters macrophage NF-κB protein synthesis resulting in repression of macrophage cell proliferation and pathogen-induced apoptosis (Ben-Abdallah et al., 2012). Purified capsule polysaccharides can trigger apoptosis of both macrophages and T-cells, which reduces the secretion of pro-inflammatory mediators from these cells (Pericolini et al., 2006; Chiapello et al., 2008; Monari et al., 2008; Villena et al., 2008). Furthermore, GXM has the ability to directly inhibit T-cell proliferation and responses (Yauch et al., 2006). Intracellular *C. neoformans* is able to modify the polarization of macrophages by altering their cytokine profiles. As the yeasts reside in the macrophages, they work to maintain a naïve-type state of the macrophages. iNOS expression is also upregulated by the intracellular yeasts (Subramani et al., 2020). Therefore, *Cryptococcus* has the insidious ability to hide within the host by manipulating the cytokine environment of the host.

Host surfactant proteins A and D (SP-A, SP-D) are an important part of the innate immune response; they bind to microbial surfaces and modulate leukocyte functions (Crouch and Wright, 2001). SP-D can bind purified GXM with high affinity but has a lower affinity for whole cryptococcal capsule, which is interesting considering GXM is the primary component of capsule (Van De Wetering et al., 2004). In cryptococcal infection, SP-A does not play an important role in clearance of fungal infection, however SP-D facilitates aggregation of microorganisms, increasing mucociliary clearance as well as promoting phagocytosis and killing in phagocytic cells. However, binding of *C. neoformans* by SP-D can also inhibit this process, increasing fungal burden and promoting systemic dissemination (Van De Wetering et al., 2004). Interestingly, SP-D is found at higher levels in the lungs of patients with AIDS, possibly contributing to the progression of disease amongst this group (Jambo et al., 2007). GXM prevents SP-D mediated aggregation which would typically facilitate pathogen clearance. SP-D may also selectively bind secreted capsule over attached capsule thereby reducing the potential for opsonization of attached capsule and subsequent immune interaction with yeast cells (Van De Wetering et al., 2004). Paradoxically, fungi bound with SP-D are protected from macrophage destruction and have increased survival (Geunes-Boyer et al., 2009; Geunes-Boyer et al., 2012). In these instances, *Cryptococcus* provides an excellent example of a pathogen using host defenses to its advantage.

Changes in capsular structure that occur during infection are necessary for transmigration into the CNS and are visible *via* immunofluorescence as soon as six hours post-intravenous inoculation (Charlier et al., 2005). Counterintuitively, an intriguing study of capsular mutants showed that a *C. neoformans* hypercapsular mutant was less virulent and showed lower levels of brain dissemination than a hypocapsular mutant (Pool et al., 2013). It has been hypothesized that capsule size is indicative of starvation stress in *Cryptococcus* (Casadevall et al., 2019), therefore neurotropism may result from the nutritionally richer environment of the deep tissues as compared to the lungs. In contrast to this hypocapsular mutant data, acapsular strains are easily phagocytosed and destroyed by immature dendritic cells and result in the upregulation of antigen-presenting molecules, showing the necessity of capsule in virulence and survival of *C. neoformans* (Vecchiarelli et al., 2003).

Capsule attachment has been recently shown to be regulated by Rim101, a transcription factor primarily involved in pH response. GXM is able to be secreted in a *rim101Δ* mutant, but the capsule is much thinner, producing a hypocapsular strain. In a mouse inhalation model, the *rim101Δ* mutant was surprisingly hypervirulent, resulting in decreased mouse survival compared to wild-type and *rim101Δ* + *RIM101* strains (O’meara et al., 2010). The *rim101Δ* strain was shown to produce a very different immune response than the wild-type strain. There was a much greater influx of inflammatory infiltrate in the lungs of the *rim101Δ* mutant, and additionally the cells composing the infiltrate differed from wild-type. The infiltrate consisted more heavily of neutrophils and eosinophils in the *rim101Δ* mutant-infected lungs, whereas in wild-type infection, the inflammatory cells mainly consist of lymphocytes and monocytes, with few neutrophils and eosinophils (O’meara et al., 2013). Cytokine levels and profiles differed as well: *rim101Δ* had higher cytokine levels than wild-type, with IL-12, IFN-γ-inducible protein 10 (IP-10), VEGF, and TNF-α all significantly increased with *rim101Δ* infection (O’meara et al., 2013).

Expression of mannoproteins MP98 and MP88 was also increased in the *rim101Δ* strain, suggesting that the altered capsule regulation correspondingly impacts the antigen profile of *C. neoformans*. Mannoproteins are structural components in the inner cell wall of *C. neoformans* and also play an antigenic role (Vartivarian et al., 1989). They are known to be the predominant antigens involved in activating cell-mediated immunity in cryptococcosis (Murphy, 1988). MP98 and MP88 are known to stimulate T-cell responses in the host reaction to *Cryptococcus* (Levitz et al., 2001; Huang et al., 2002), which may at least partially explain the hypervirulence phenotype in this mutant.

### 2.2 Cell Wall

#### 2.2.1 Composition and Immune Interaction

The cell wall of *Cryptococcus* is a two-layered structure that sits above the plasma membrane and serves to control cellular permeability in addition to mitigating mechanical and osmotic stresses (Baker et al., 2007; Ponton, 2008; Doering, 2009; O’meara and Alspaugh, 2012; Gow et al., 2017; Agustinho et al., 2018; Wang et al., 2018). In fungi, the cell wall is composed of glucans, chitin, chitosan, glycosylated proteins, and melanin (Casadevall and Perfect, 1998). Glucans are the most important structural polysaccharides of the cell wall and are synthesized in the plasma membrane by a series of enzymes called glucan synthases. The inner layer is mostly fibers of β-glucan and chitin arranged parallel to the plasma membrane, while the outer layer contains α-glucan and β-glucan (Sakaguchi et al., 1993; Doering, 2009; O’meara and Alspaugh, 2012). Alpha-1,3-glucan is required to anchor the capsule to the cell wall, and is required for virulence (Reese and Doering, 2003; Reese et al., 2007).

Chitin is synthesized by an enzyme called chitin synthase from N-acetylglucosamine (GlcNAc) and is deposited into the extracellular space next to the plasma membrane. In *Cryptococcus* species, chitin is a minor component of the cell wall but it does contribute strength and plays a crucial role in capsular architecture (Doering, 2009; Zaragoza et al., 2010). Chitin also has the ability to modulate host immune responses (Wiesner et al., 2015). The deacetylated form of chitin, chitosan, is more soluble and flexible than chitin, making up 3-5 times more of the cell wall (by dry weight) than chitin and is necessary for cryptococcal virulence (Upadhya et al., 2016). As the density of the cell wall changes, so does the ratio of chitosan to chitin (Banks et al., 2005). Chitosan is important for maintaining the integrity of the cell wall and normal capsule width; mutants deficient in chitosan display a “leaky” phenotype and are unable to retain melanin within the cell wall (Baker et al., 2007).

Changes to antigenic structures due to alterations in carbon metabolism have been elucidated in other pathogenic fungi. In *Candida albicans*, glucose is preferred carbon source, but alternative non-fermentable carbon sources such as lactate, acetate and citrate can be assimilated through gluconeogenesis, glyoxylate shunt, and β-oxidation. Interestingly, growth of *Candida* on alternative carbon sources also modifies the composition and architecture of the cell wall, resulting in altered host recognition and immune cell interactions (Ene et al., 2012; Ene et al., 2013). When grown in host-derived blood or serum, *Candida* exhibited a dramatic reduction in the length of mannan chains, as well as a reduction in cell wall structural complexity (Lowman et al., 2011). With lactate as a sole carbon source, biomass was significantly reduced and cell walls were thinner, with the β-glucan and chitin layer dramatically reduced. Although these components maintained the same proportions, these cells displayed alterations to cell wall porosity and hydrophobicity (Ene et al., 2012), both of which could potentially affect pathogenicity. The observed changes result in decreased host recognition and phagocytosis, while those yeasts that are engulfed show increased macrophage killing and escape (Brown et al., 2014). This phenotype results in increased virulence and fungal burden and the promotion of a non-protective M2 phenotype as indicated by the increased secretion of IL-10 (Ene et al., 2012; Ene et al., 2013) (**Figure 1**). Since many of the cell wall components are recognized by pattern-recognition receptors on antigen presenting cells, alterations in the cell wall structure may result in the inability of PAMPs to bind their antigenic targets and generate a protective immune response (Lewis et al., 2012).

## 3 Carbon Sources at Sites of Infection

*C. neoformans* can rapidly acclimate to environments with variable nutrient sources, allowing it to grow and proliferate within not only the external environment, but also the many different environments within the host. Pathogen fitness and virulence is directly related to nutrient acquisition and metabolism, particularly the utilization of carbon (Ramachandra et al., 2014). *Ex vivo*, *Cryptococcus* lives in detritus and bird feces, both of which have readily available carbon and nitrogen sources (Barkal et al., 2016). As pathogenic yeasts move into a mammalian host, they must find alternate ways of providing the energy and substrates needed for cellular processes (Barelle et al., 2006). Nutrient availability and environmental conditions are continually shifting, and vary by early and late infection, as well as by tissue type during dissemination (Hu et al., 2008). In fact, identical clinical isolates (based on multi-locus sequence typing) from an initial and relapse infection were shown to have significant phenotypic differences in metabolic profiles and dissemination patterns while maintaining similar levels of virulence (Ormerod and Fraser, 2013). This suggests that specific host-generated stresses, such as alterations to carbon source availability within a tissue, result in gene expression changes that are maintained clonally amongst cells within an isolated environment and persist in long-term infection. The primary carbon metabolism pathways influencing virulence of *C. neoformans* within the host include glycolysis, gluconeogenesis, β-oxidation, the tricarboxylic acid (TCA) cycle and the glyoxylate shunt (Cherniak et al., 1998a). In yeasts, peroxisomes are important for catabolic metabolic functions including β-oxidation and the glyoxylate shunt (Sibirny, 2016). Utilization of monosaccharides such as fructose, glucose and mannan requires peroxisomal processing (Idnurm et al., 2007). Many of the products and intermediates of carbon metabolism are utilized for the generation of structural features such as the cell wall and capsule (Wang et al., 2018).

The presence of glucose blocks the usage of alternate carbon sources due to a regulatory process called carbon catabolite repression. In order to transition from glucose metabolism to alternate sources, there must be activation of the protein kinase Snf1, a regulator of the carbon catabolite repressor Mig1 (Hu et al., 2008). This pathway is especially important in the CNS, where glucose is present in higher concentrations compared to what is experienced in the lungs or in the blood. Another transcription factor repressed by the presence of glucose is Nrg1, a downstream effector in the cAMP pathway, which has been shown to regulate many genes associated with metabolism and transport of carbohydrates. In addition to its role in energy production, Nrg1 appears to show some regulation of capsule production and cell wall integrity (Cramer et al., 2006). *C. neoformans* cells are subjected to glucose deprivation within the body and must utilize alternate carbon sources (Panepinto et al., 2005; Hu et al., 2008; Price et al., 2011) which is done through the upregulation of oxidative phosphorylation (Chun et al., 2007; Ingavale et al., 2008).

### 3.1 Respiratory System

Genes associated with lipid degradation and fatty acid catabolism are elevated at 8 hours (Hu et al., 2008), consistent with localization of yeasts in the alveoli, where phospholipid-rich surfactant is an abundant carbon source. Surfactant is primarily composed of phospholipids, with phosphatidylcholine making up about 80% of the lipids, and phosphatidylglycerol making up 7-15% of the lipid content. Phosphatidylcholine is the surface-active component, while phosphatidylglycerol is thought to play a role in immune response. Surfactant also contains proteins involved in regulation of the structure and properties of the lipid film, while others are required for the innate immune response and inflammation (Agassandian and Mallampalli, 2013).

The expression of genes associated with lipid degradation and fatty acid catabolism decreases by 24 hours as the yeasts move into the tissues (Rooney et al., 1994; Feldmesser et al., 2000; Fan et al., 2005). Lipids are also available intracellularly within macrophages, which may serve as a carbon source during intracellular parasitism. The secreted enzyme phospholipase B1 (*PLB1*) is able to liberate carbon from phospholipids found in both surfactant and cell membranes, including those of the phagolysosome helping to promote fungal escape (Wright et al., 2007). Activity of this enzyme allows for the metabolism of host-derived fatty acids, but also allows for the incorporation of these lipids into cellular products. One such metabolite, arachidonic acid, is not naturally found in cryptococci but is obtained from macrophages after phagocytosis (Wright et al., 2007) and can then be used to produce compounds with immunomodulatory affects such as the oxylipin prostaglandin E2 (PGE2) (Erb-Downward and Huffnagle, 2007).

The glyoxylate cycle is an anapleurotic variant of the TCA cycle and an anabolic process that allows cells to metabolize 2-carbon compounds (e.g. acetate and ethanol) when simple sugars are not available (Mccammon, 1996; Kanai et al., 1998), and its expression has been shown to be enhanced in conditions of low glucose (Wayne and Lin, 1982) (**Figure 1**). Since carbon is already deficient in a host environment, this pathway allows for energy production without the unnecessary loss of elemental carbon by utilizing a shunt to bypass some of the traditional steps of the TCA cycle (Hu et al., 2008; Dunn et al., 2009). Unlike other carbon utilization pathways, the glyoxylate shunt is not required for virulence in *C. neoformans* as it is in other pathogenic organisms (Lorenz and Fink, 2001; Rude et al., 2002; Idnurm et al., 2007). Isocitrate lyase (Icl1) is the primary enzyme controlling the glyoxylate shunt pathway (Fernández et al., 1992; Barth and Scheuber, 1993; Umemura et al., 1997). In a human host, levels of Icl1 and another key enzyme, malate synthase (Mls1), are increased and elimination of these enzymes resulted in an inability of the yeasts to grow on acetate as a sole carbon source (Idnurm et al., 2007). Aconitase and succinate dehydrogenase, enzymes of the TCA and glyoxylate cycles, were also upregulated in early lung infection (Hu et al., 2008).

### 3.2 Central Nervous System

Dissemination from the lungs to other organs results in exposure to different nutrient pools and consequently the requirement for metabolic adaptation in each new environment (Guess et al., 2018). Glycolysis is the preferred metabolic pathway within the CNS, however glucose is not always available due to the high metabolic demand from CNS cells. Although gluconeogenesis is not the primary method of carbon acquisition within the CNS, *PCK1* (phosphoenolpyruvate carboxykinase) expression is upregulated in the low glucose concentrations of CSF (Price et al., 2011) (**Figure 1**). Additionally, several studies have indicated that expression of *ICL1* is also induced as a result of significant decreases in CSF glucose concentrations during cryptococcal meningoencephalitis, promoting utilization of the glyoxylate shunt for carbon assimilation (Perfect et al., 1980; Kwon-Chung et al., 2000; Rude et al., 2002). Ethanol and acetate have been shown to be available in brain tissue and the subarachnoid space as substrates for the glyoxylate shunt after their conversion to acetyl-CoA (Chew et al., 2019). While these pathways are certainly utilized, deletion mutant studies of *PCK1*, *SNF1* and *ACS1* (acetyl-CoA synthetase) suggest that fungal persistence and disease production within the brain is likely due to carbon assimilation *via* glycolysis (Hu et al., 2008; Price et al., 2011).

Carbon assimilation *via* glycolysis in the CNS is supported by the characterization of two glycolysis mutants with significantly decreased persistence within the CNS: a pyruvate kinase mutant (*pyk1Δ*) and a hexokinase double-mutant (*hxk1Δ/hxk2Δ*) (Price et al., 2011) (**Figure 2**). Apparently, blocking either the entry of substrates into or exit of substrates from the glycolytic pathway has severe metabolic consequences impacting yeast survival within the CNS. Additionally, access to oxygen is necessary for oxidative metabolism and hypoxic conditions within the brain tissue require cryptococcal yeasts to colonize the more highly vascularized areas (mainly gray matter) after crossing the blood-brain barrier in order to maintain aerobic respiration (Chang et al., 2007). Oxygen sensing mechanisms are required for the appropriate transcriptional responses to a low-oxygen environment, and upon activation alter essential processes in energy metabolism such as mitochondrial function and associated carbohydrate metabolism such as the electron transport chain (oxidative phosphorylation) (Chang et al., 2007; Ingavale et al., 2008).

## 4 Conclusion

**Figure 3:**
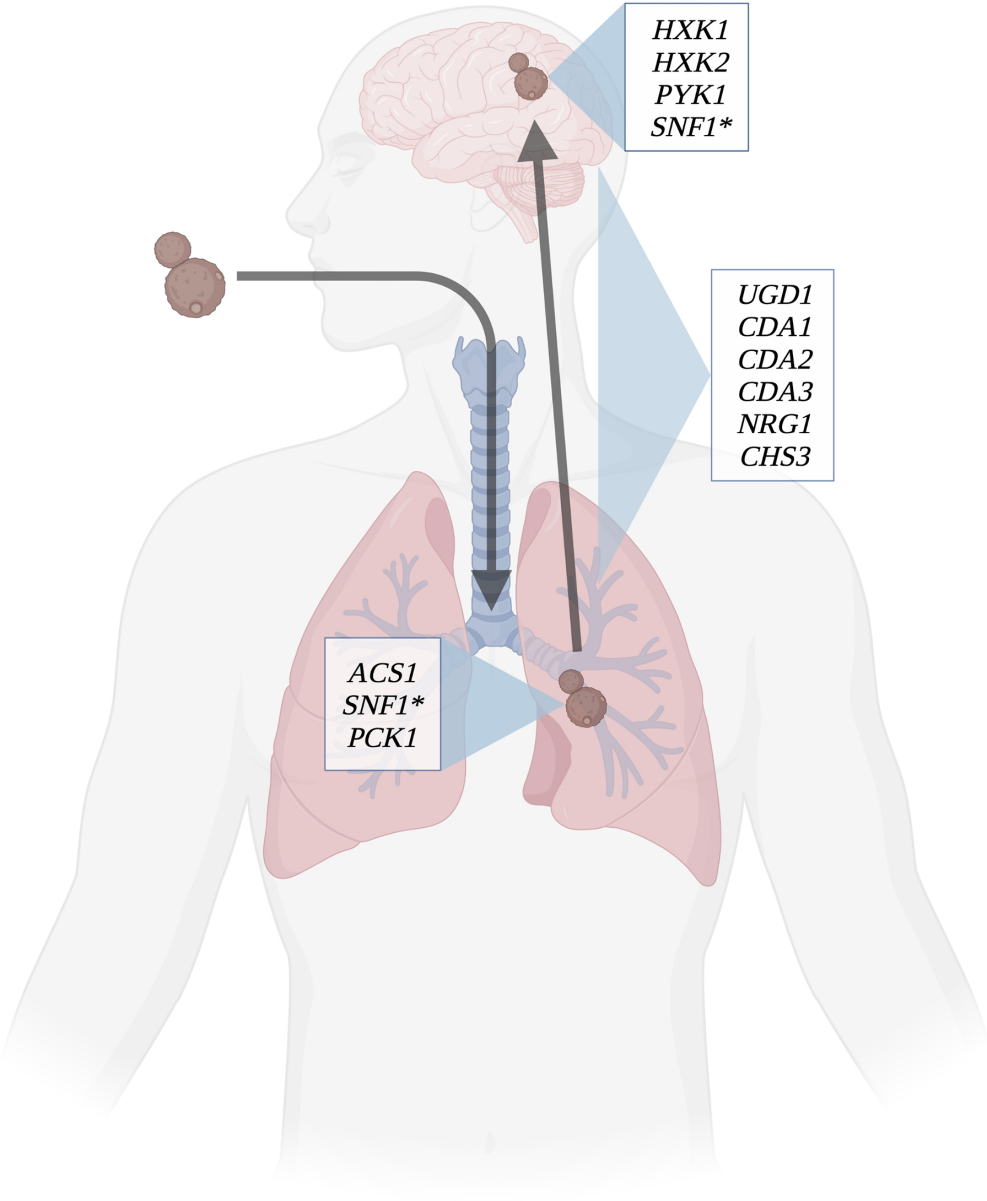
Determinants of virulence throughout infection course. Several key genes involved in carbon metabolism have been shown to affect virulence in *C. neoformans*. Certain genes involved in capsule and cell wall synthesis are expressed throughout the course of infection; these include chitin synthases, UDP-glucose dehydrogenase, and capsule regulation genes. *ACS1* and *PCK1* are essential to virulence in lung infection, as they allow *C. neoformans* to use the lung environment to make capsule and cell wall. *HXK1*, *HXK2*, and *PYK1* are involved in using cerebrospinal fluid as a carbon source for the yeasts to thrive. SNF1(*) is utilized in both the lung and brain environments to regulate carbon metabolism. Created with Biorender.com.

While generally considered a fitness attribute and not a virulence factor, the ability to use multiple carbon sources is essential for compatible interactions of pathogenic *Cryptococcus* species with their hosts (**Figure 3**). In addition to being utilized for energy production, carbon metabolism produces several substrates and products that are incorporated into immunogenic components such as the cell wall and capsule. Although *Cryptococcus* is somewhat unique in its production of polysaccharide capsule, other immunogenic structures such as glucans and chitin within the cell wall can also be affected. Impairing the production of these substrates has been shown to result in various virulence defects (Moyrand et al., 2002), but relatively few direct connections have been made between defects in pathogen carbon metabolism and changes in host immune responses to this organism. Although lacking a polysaccharide capsule, *C. albicans* shares many of the pathways for cell wall production with *Cryptococcus*, therefore similar changes to immune recognition observed in *Candida* in response to altered carbon metabolism can serve as a guide to these responses in *Cryptococcus*. As one of the primary virulence factors in *Cryptococcus*, production of capsule is dependent on the availability of sugar groups for polysaccharide synthesis. Capsule morphology has been shown to change in response to nutrient availability in host pathogenesis resulting in alterations to capsule antigenicity as yeasts disseminate systemically. Understanding what responses these carbon metabolites of *Cryptococcus* elicit from the host immune system will further illuminate the mechanisms of disease, and also the processes of other outcomes (e.g. fungal clearance vs. dormancy; **Figure 1**). The exact structural changes that occur and their role in immune recognition are fertile ground for future research in this important emerging fungal pathogen.

## Author Contributions

HB wrote the manuscript. LC wrote parts of the manuscript. MP edited and wrote parts of the manuscript. All authors contributed to the article and approved the submitted version.

## Funding

MP was supported in part by the Liberty University Department of Biology and Chemistry, and grants from the Liberty University Center for Research and Scholarship and the Liberty University College of Osteopathic Medicine Center for Research. LC was supported by the Liberty University Department of Biology and Chemistry. HB was supported by the Liberty University College of Osteopathic Medicine Anatomy Teaching Fellowship.

## Conflict of Interest

The authors declare that the research was conducted in the absence of any commercial or financial relationships that could be construed as a potential conflict of interest.

## Publisher’s Note

All claims expressed in this article are solely those of the authors and do not necessarily represent those of their affiliated organizations, or those of the publisher, the editors and the reviewers. Any product that may be evaluated in this article, or claim that may be made by its manufacturer, is not guaranteed or endorsed by the publisher.
